# Japanese encephalitis virus and its mechanisms of neuroinvasion

**DOI:** 10.1371/journal.ppat.1008260

**Published:** 2020-04-02

**Authors:** Justin T. Hsieh, Ashley L. St. John

**Affiliations:** 1 Program in Emerging Infectious Diseases, Duke-National University of Singapore Medical School, Singapore; 2 Department of Microbiology and Immunology, Yong Loo Lin School of Medicine, National University of Singapore, Singapore; 3 SingHealth Duke-NUS Global Health Institute, Singapore; 4 Department of Pathology, Duke University Medical Center, Durham, North Carolina, United States of America; Mount Sinai School of Medicine, UNITED STATES

## Introduction

Japanese encephalitis virus (JEV) is a positive-sense single-stranded RNA virus of the *Flavivirus* genus that is spread by *Culex* mosquitos. It is maintained in an enzootic cycle in pigs and wild birds in which humans are dead-end hosts [1]. Despite having effective vaccines, JEV is the leading cause of viral encephalitis in Asia [1]. As a neuroinvasive virus, it can effectively cross the blood–brain barrier (BBB) to cause acute encephalitis. Twenty-five percent to 30% of Japanese encephalitis (JE) cases are fatal, and 50% result in permanent neuropsychiatric complications [2]. There are currently no treatments for JE, partly due to an incomplete understanding of the mechanisms promoting encephalitis.

The central nervous system (CNS) relies on the BBB, a tightly regulated barrier between the peripheral circulation and the CNS, to prevent entry of pathogens, including viruses. Yet, JEV and other neuroinvasive viruses can overcome the BBB, which usually excludes foreign substances. It is formed primarily by tight junctions (TJ) between endothelial cells, comprised of proteins such as claudin-5, zonula occludens (ZO)-1, and occludin. The BBB is sustained by supporting cell types, including astrocytes, pericytes, microglia, and mast cells (MCs) [1, 3]. Together, these cells form neurovascular units that maintain a barrier along the cerebrovascular microvessels to promote immune privilege and CNS homeostasis [3, 4].

**Fig 1:**
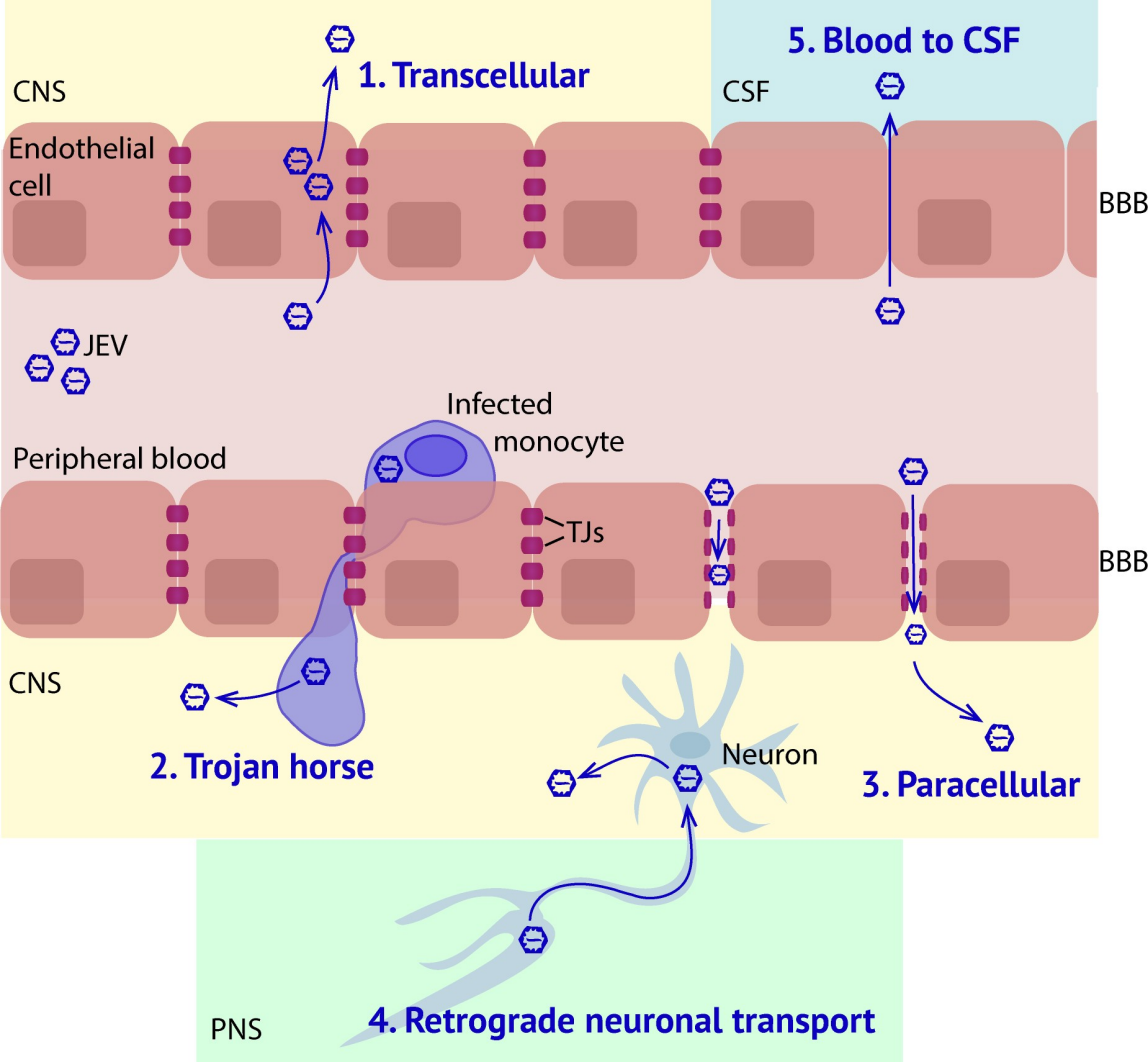
Hypothetical mechanisms of viral entry into the CNS. Diagram depicting 5 mechanisms through which viruses can enter the CNS. (1) Transcellular transport within endothelial cells of the BBB through transcytosis or infection and release of viral particles into the CNS. (2) Passage through the BBB within infected cells such as monocytes in a “Trojan Horse” method. (3) Paracellular trafficking of virus across the BBB at locations where TJs have been cleaved, resulting in permeability. 4) Retrograde neuronal transport where CNS neurons that contact the PNS become infected. 5) Inoculation of the CSF at locations where endothelial cells lack BBB function, such as in circumventricular organs. BBB, blood–brain barrier; CNS, central nervous system; CSF, cerebral spinal fluid; JEV, Japanese encephalitis virus; PNS, peripheral nervous system; TJ, tight junction.

Neuroinvasive viruses use several mechanisms to access the CNS: (1) direct infection of endothelial cells and subsequent transcellular release of virus into the brain parenchyma, (2) infection of peripheral immune cells that enter the CNS in a “Trojan Horse” mechanism, (3) paracellular entry following breakdown of the BBB, (4) retrograde transport of virus from the peripheral nervous system (PNS) into the CNS, and (5) translocation from the blood to the cerebral spinal fluid (CSF) [5, 6] (Fig 1). JEV infection through the natural subcutaneous route leads to widespread infection in various parts of the brain [7], suggesting a hematological route of infection, such as would occur for mechanisms 1–3. Here we provide an overview of a few described mechanisms of JEV penetration of the BBB and processes that amplify CNS infection.

## Transcellular infection of endothelial cells and activation of the neurovascular unit

Some neurotropic viruses directly infect endothelial cells to reach the brain from the circulation and travel transcellularly to release viruses into the brain parenchyma [6]. JEV has been visualized intracellularly in vesicles using electron microscopy and was suggested to undergo transcytosis across endothelial cells through pericytes into the brain of infected suckling mice [3]. JEV antigens have not been observed in brain endothelial cells in virus-infected adult mice[8] or consistently observed in brain specimens from fatal JE patients [9], but this mechanism of neuroinvasion could potentially contribute to CNS infection. Cell-culture studies have also shown that endothelial cells may transiently propagate JEV to other supporting cells such as astrocytes, which can subsequently be activated [10].

## Paracellular infection is promoted by MCs

**Fig 2:**
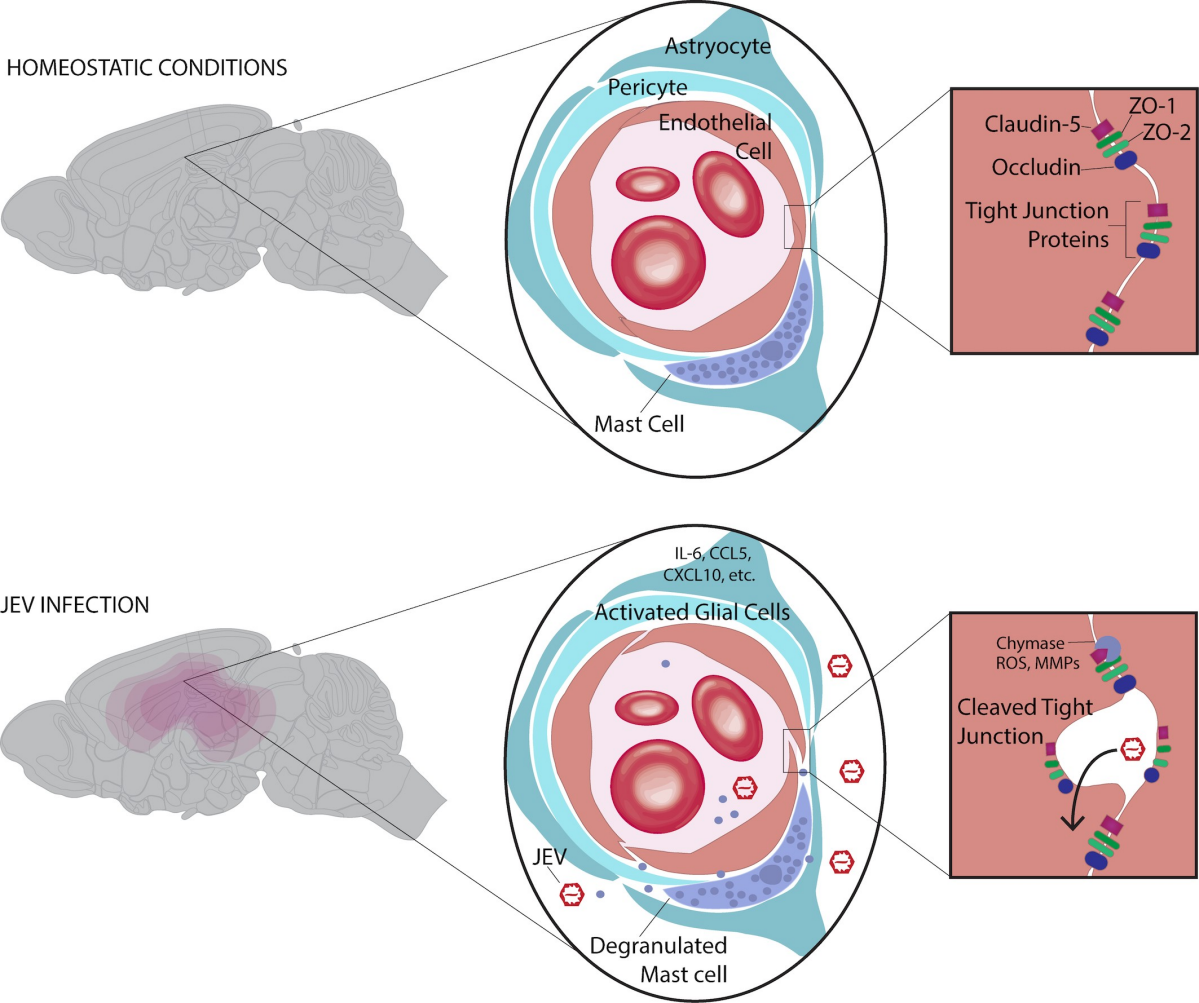
MC chymase induces BBB breakdown and paracellular JEV entry into the CNS. JEV activates MCs, leading to degranulation and the release of MC-derived chymase. Chymase cleaves brain endothelial TJ proteins ZO-1, ZO-2, claudin-5, and occludin, compromising the BBB and facilitating JEV penetration into the CNS. Other cells in the neurovascular unit such as astrocytes and pericytes are also activated during JEV infection and release proinflammatory cytokines. BBB, blood–brain barrier; CNS, central nervous system; JEV, Japanese encephalitis virus; MC, mast cell; MMP, matrix metalloproteinase; ROS, reactive oxygen species; TJ, tight junctions.

Neurotropic viruses can gain entry into the brain through compromising the BBB. This occurs when TJs between endothelial cells become leaky, often due to the proinflammatory response. That JEV gains entry into the brain through cleaved TJs is supported by reduced resistance across JEV-exposed cultured brain-endothelial-cell membranes in vitro [11] and increased BBB leakiness and TJ protein breakdown in mouse models [12]. MCs are an important population of CNS-resident immune cells recently implicated in BBB compromise during JEV infection [12]. Indeed, MC depletion from the CNS reduced BBB breakdown and JEV penetration of the brain in mice [12]. In addition to their presence within the brain, MCs are also one of the first immune cell types JEV encounters in the periphery. JEV causes MC degranulation, which enhances JEV-induced breakdown of the BBB and augments infection in the brain [13]. In particular, MC-derived chymase, a vasoactive protease, played a functionally significant role in breakdown of TJ proteins, including ZO-1, ZO-2, claudin-5, and occludin, ultimately leading to increased BBB compromise (Fig 2). This was restored with either pharmacological inhibition of chymase or genetic knockout of the mouse-chymase MCPT4 [2]. In vivo, therapeutically inhibiting chymase reduced JEV penetration of the BBB and reduced the morbidity and mortality associated with JE [12]. Thus, MCs facilitate paracellular entry of JEV across the BBB.

## Other peripheral myeloid cells may contribute to neuroinvasion

Another potential pathway establishing CNS infection is through infection of peripheral immune cells that can subsequently gain entry into the CNS in a “Trojan Horse” mechanism. For JEV infection, macrophages [11] and DCs [14] are suspected peripheral infection cell targets. Furthermore, macrophages are the predominant infiltrated immune cells in fatal JEV brain samples [13]. However, in vivo experiments, thus far, have failed to identify infected macrophages in CNS tissues at the time points tested [11]. This does not rule out the possibility of low titer introduction of a virus into the CNS in this manner, but further investigation is needed. Alternatively, peripheral macrophages may indirectly promote neuroinvasion through the release of other mediators, such as reactive oxygen species (ROS) and MMP-9 [13] (Fig 2). In contrast to the suspected role of macrophages in BBB compromise during JE, JEV-infected DCs promote anti-inflammatory regulatory T cells (T-regs) while inhibiting proinflammatory Th-17 T cells and monocyte differentiation [15, 16]. These modulations are thought to result in improved BBB integrity and reduced viral entry into the CNS, demonstrating the dual contributions of peripheral immune responses to BBB integrity and infection control.

## Amplification of BBB leakiness by JEV infection within the brain

Once present in the CNS, inflammation due to JEV amplifies BBB breakdown. Pericytes, which are situated within the basement membrane next to endothelial cells, aid in breakdown of the BBB through release of IL-6, which leads to ZO-1 degradation [17]. Coculture of astrocytes and brain endothelial cells increases TJ leakiness through increased release of mediators such as IL-6, CCL5, and CXCL10 [17] (Fig 2). Microglia also release proinflammatory mediators such as TNF-α to promote BBB leakiness [17]. At the latter stages of disease, CNS supporting cells are activated and accentuate BBB leakiness and disease, but this increased permeability may also allow penetration of immune cells that are necessary for infection clearance, such as T cells [18].

## Dual role of interferons in JEV protection and BBB permeability

Another factor that has been implicated in accentuating BBB breakdown during JEV infection are interferons (IFNs). Quick induction of type I IFNs has been associated with protection of astrocytes from apoptosis [19] and protection from lethal infection in mice [20]. Similarly, IFNγ was shown to contribute to protection from CNS infection [18]. In contrast, targeting of IFNγ with a neutralizing antibody improved BBB integrity in one study, suggesting its role in enhancing BBB permeability [21].

## Concluding remarks

JEV causes high morbidity and mortality in humans, leading to permanent neurological deficits, even in those who survive. Recent reports have advanced our understanding of the pathophysiological events that allow JEV to traverse the BBB and cause encephalitis. Although multiple routes of CNS entry are plausible, paracellular penetration of viruses resulting from protease and cytokine-driven breakdown of the BBB appears to be the dominant mechanism for JEV neuropenetration. This new knowledge may aid to the development of therapeutics for the treatment of virally induced encephalitis.
